# Interfacial Structural Transformation for the Synthesis of Lead‐Free Double Perovskite Nanocrystals

**DOI:** 10.1002/advs.202416046

**Published:** 2025-03-08

**Authors:** Jun Liu, Anna A Vedernikova, Qi Xue, Huiying Gao, Xiuhui Xie, Jinfeng Xie, Elena V Ushakova, He Huang, Xiaohong Zhang

**Affiliations:** ^1^ School of Optoelectronic Science and Engineering & Collaborative Innovation Center of Suzhou Nano Science and Technology Soochow University Suzhou 215006 P. R. China; ^2^ Key Lab of Advanced Optical Manufacturing Technologies of Jiangsu Province &, Key Lab of Modern Optical Technologies of Education Ministry of China Soochow University Suzhou 215006 P. R. China; ^3^ International Research and Education Centre for Physics of Nanostructures ITMO University Saint Petersburg 197101 Russia; ^4^ School of Physical Science and Technology Soochow University Suzhou 215006 P. R. China; ^5^ Department of Materials Science and Engineering and Centre for Functional Photonics (CFP) City University of Hong Kong Hong Kong SAR 999077 P. R. China; ^6^ Institute of Functional Nano and Soft Materials (FUNSOM) Jiangsu Key Laboratory for Carbon‐Based Functional Materials and Devices Soochow University Suzhou 215123 P. R. China

**Keywords:** double perovskite, lead‐free, post‐synthetic method, structural transformation

## Abstract

Lead‐free halide double perovskite nanocrystals have emerged as one of the most promising alternatives to lead halide perovskite nanocrystals due to their non‐toxicity, high stability, and outstanding optoelectronic properties. However, conventional synthesis methods often result in impurities due to increased constituent elements. In this study, an efficient water‐oil biphasic interface‐driven approach is introduced for synthesizing lead‐free double perovskite nanocrystals, enabling controlled structural transformations from 0D to 2D and 3D structures. Starting from 0D Cs_3_BiBr_6_, a gradual cation exchange is achieved, forming 3D Cs_2_AgBiBr_6_. Real‐time monitoring reveals the slow insertion of Ag^+^ ions as the key to the structural transformation. The resulting Cs_2_AgBiBr_6_ nanocrystals exhibit exceptional stability, maintaining their integrity for over 120 days under ambient conditions without significant degradation, showing no considerable material decomposition. Additionally, this method allows for the successful synthesis of 2D layered double perovskite Cs_4_ZnBi_2_Br_12_, which has not previously been reported in experimental studies. This biphasic synthesis strategy provides a universal and reliable method for producing high‐quality double perovskite nanocrystals while offering valuable insights into their structural dynamics and properties.

## Graphical abstract

The water‐oil biphasic method facilitates the structural transformation of 0D perovskites Cs_3_BiBr_6_ into 3D double perovskites Cs_2_AgBiBr_6_ and 2D layered double perovskites Cs_4_ZnBi_2_Br_12_.The water phase removes excess impurities, enhancing crystallinity and stability through a controlled interfacial reaction, crucial for advanced optoelectronic applications.

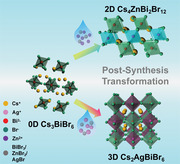

## 1 Introduction

Lead halide perovskites are widely recognized for their excellent photoelectric properties and outstanding performance in optoelectronic devices.^[^ ^1^ ^]^ However, the toxicity and inherent instability of lead halide perovskites pose significant challenges to their widespread adoption. To address this concern, the exploration of non‐toxic alternatives, preserving the outstanding optoelectronic characteristics of lead‐based perovskite nanocrystals (NCs), has become a critical area of research. Lead‐free double perovskites (DPs) have emerged as promising alternatives to lead halide perovskites because of their high stability and low toxicity.^[^ ^2^ ^]^ The general chemical formula of lead‐free DPs is A_2_B^+^B^3+^X_6_, where monovalent and trivalent metal cations replace the toxic lead ions in the crystal structure.^[^ ^3^ ^]^ Furthermore, layered double perovskites (LDPs) with a general chemical formula of A_4_B^2+^B^3+^_2_X_12_, have drawn attention due to their unique layered structures, which further diversify the potential applications of DPs.^[^ ^4^ ^]^ High‐quality lead‐free DP NCs have been widely applied in various fields, including light‐emitting diodes,^[^ ^5^ ^]^ ultraviolet and X‐ray detectors,^[^ ^6^ ^]^ and solar cell devices.^[^ ^7^ ^]^

In recent years, extensive efforts have been devoted to developing various synthetic strategies for DP NCs of different compositions and morphologies to optimize their crystalline quality. Most of these methods for synthesizing DP NCs employ direct synthetic routes, with the hot‐injection method being particularly favored.^[^ ^8^ ^]^ However, due to the involvement of multiple elements in the reaction, direct synthesis at high temperatures may lead to the generation of side products.^[^ ^9^ ^]^ This has driven the exploration of alternative, indirect synthetic approaches to overcome these limitations.

Herein, we introduce a water‐oil interfacial strategy for the efficient synthesis and controlled structural transformation of DP NCs, exemplified by the conversion of 0D Cs_3_BiBr_6_ into 3D Cs_2_AgBiBr_6_ and 2D Cs_4_ZnBi_2_Br_12_. Distinct from conventional approaches, our biphasic system employs immiscible phases to foster a stable and controlled environment for the synthesis of perovskite NCs. The aqueous phase, in which high concentrations of metal salts are dissolved, ensures meticulous control over the ionic concentrations, thus facilitating precise ionic delivery. Concurrently, the nonpolar toluene phase stabilizes the growing perovskite NCs, preserving their quality and structural integrity. Moreover, ligands play a pivotal role at the interface, acting as molecular bridges that enhance the transfer of ions between phases and ensure uniform nucleation. This water‐oil biphasic approach not only allows for real‐time monitoring of the NCs transformation but also sheds light on the mechanisms of cation exchange and the structural evolution inherent in these materials. Notably, the resulting DP NCs exhibit remarkable stability, maintaining their structure for over 120 days under ambient conditions (room temperature and ambient light conditions, without any controlled humidity or atmosphere). This universal and robust biphasic strategy expands the synthetic toolkit for perovskite materials, offering new possibilities for optoelectronic applications by overcoming the limitations of conventional methods.

## 2 Results and Discussion

**Scheme 1:**
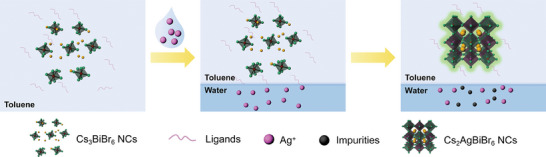
Schematic illustration of the transformation from Cs_3_BiBr_6_ to Cs_2_AgBiBr_6_ via the water‐oil biphasic method.

**Scheme** **1** illustrates the process of synthesizing high‐quality DP NCs using the water‐oil biphasic method. The introduction of AgNO_3_ through an immiscible water phase creates a well‐defined phase separation interface with toluene. The electron‐donating ligand oleylamine acts as a molecular shuttle at the water–toluene interface to transport AgNO_3_ from water to toluene.^[^ ^10^ ^]^ This distinct interface confines the reaction to the boundary, with the assistance of ligands, enabling a controlled interfacial reaction that minimizes NC damage. Moreover, the water phase serves as a sink for the byproducts, promoting effective impurity removal from the reaction system. As a result, the water‐oil biphasic method produces DP NCs with improved crystallinity, enhanced stability, and less impurities, marking a significant advancement over traditional single‐phase synthetic approaches.

**Figure 1:**
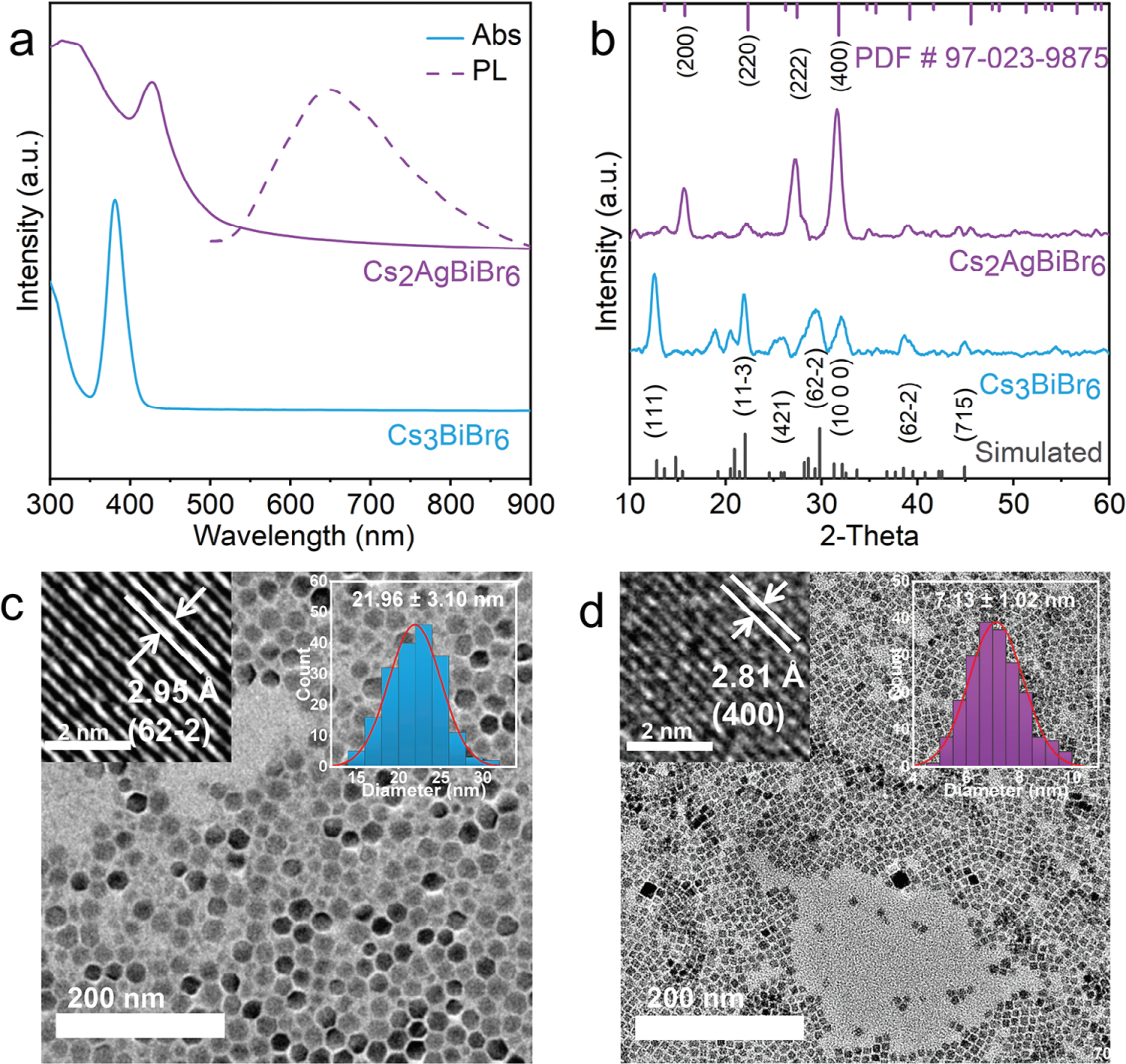
a) Absorption spectra (solid lines) and PL spectra (dashed line), b) XRD patterns, TEM images, HR‐TEM images (left inset), and particle size distribution histograms (right inset) of Cs_3_BiBr_6_ NCs (c) and Cs_2_AgBiBr_6_ NCs (d) obtained by the transformation reactions.

0D Cs_3_BiBr_6_ NCs were synthesized using a modified hot‐injection method (more details are given in Support Information). Compared with DPs, Cs_3_BiBr_6_ involves fewer elements in the reaction, allowing 0D Cs_3_BiBr_6_ NCs to be easily obtained by hot‐injection without producing other phase side products. The synthesized Cs_3_BiBr_6_ NCs exhibited a strong absorption band at 380 nm with low detectable photoluminescence (PL) signals (Figure S1a, Supporting Information). The X‐ray diffraction (XRD) pattern (Figure S1b, Supporting Information) shows numerous peaks attributed to the low symmetry of the monoclinic phases within the C2/c space group. These peaks reflect the monoclinic crystal structure of the 0D perovskite. The crystal structure of Cs_3_BiBr_6_ consists of [BiBr_6_]^3−^ octahedra, where each Bi^3+^ ion coordinates with 6 Br^−^ ions. These [BiBr_6_]^3−^ octahedra are individually separated, resulting in a 0D perovskite structure.^[^ ^11^ ^]^ The lattice parameters were calculated as a = 28.31 Å, b = 8.617 Å, c = 13.71 Å, β = 99.48° (Table S1, Supporting Information).^[^ ^11c^ ^]^ Transmission electron microscopy (TEM) measurements reveal that the Cs_3_BiBr_6_ NCs possess hexagonal shape with an average diameter of 21.96 ± 3.10 nm (**Figure** **1**c; Figure S2, Supporting Information).

The pure phase Cs_2_AgBiBr_6_ can be easily obtained by using high‐quality Cs_3_BiBr_6_. The 3D DP Cs_2_AgBiBr_6_ was formed by the structural transformation of the 0D Cs_3_BiBr_6_ perovskite upon the addition of a certain amount of AgNO_3_ aqueous solution (more details are given in Support Information). Similarly, using Cs_3_BiCl_6_ under comparable conditions allowed for the successful synthesis of Cs_2_AgBiCl_6_, demonstrating the universality of this method for different halide perovskites (Figure S3, Supporting Information). The optical and morphological properties of the synthesized Cs_2_AgBiBr_6_ were tested to demonstrate the complete conversion of the precursor into Cs_2_AgBiBr_6_ (Figure 1). The absorption peak of Cs_3_BiBr_6_ at 380 nm vanished and was replaced by a new peak at 430 nm (Figure 1a). Additionally, a broad PL band was observed at 650 nm. The emission can be attributed to the self‐trapped exciton emission of the Cs_2_AgBiBr_6_ NCs.^[^ ^4b^ ^]^ The XRD pattern (Figure 1b) shows peaks in positions and relative intensity similar to those of the standard card (PDF # 97‐023‐9875), confirming the successful structural transformation from 0D Cs_3_BiBr_6_ NCs to 3D Cs_2_AgBiBr_6_ DP NCs. To further investigate the effect of Ag⁺ ion concentration in the aqueous phase on the crystallinity of the products, we conducted a series of experiments by varying the concentration of AgNO_3_. Thus, AgNO_3_ solutions at concentrations of 2 g mL^−1^ (11.77 m), 2 g/3 mL (3.92 m), 2 g/5 mL (2.35 m), and 2 g/10 mL (1.18 m) were prepared. The XRD patterns of the resulting Cs_2_AgBiBr_6_ samples are shown in Figure S4 (Supporting Information). The XRD data demonstrate a clear correlation between the Ag^+^ ion concentration in the aqueous phase and the crystallinity of Cs_2_AgBiBr_6_. At the highest Ag^+^ concentration (2 g mL^−1^), the diffraction peaks are sharp and intense, indicating high crystallinity. As the Ag^+^ concentration decreases, the XRD patterns show increasing noise, reflecting a reduction in the crystallinity. Detailed analysis of the XRD patterns confirm that higher Ag^+^ concentrations promote complete cation exchange and structural transformation from Cs_3_BiBr_6_ to Cs_2_AgBiBr_6_, resulting in improved crystal quality.

To explore the morphological and size distribution changes resulting from the structural transformation of the crystals, TEM images were collected and are presented in Figure 1c,d (corresponding high‐resolution‐TEM (HR‐TEM) images and particle size distribution histograms are shown in insets). TEM images reveal that the Cs_2_AgBiBr_6_ NCs exhibit a uniform size distribution and a clear cubic shape, with an average diameter of 7.13 ± 1.02 nm (Figure 1d, right inset). The TEM images and particle size distribution histograms of the intermediate products collected at 15, 30, and 45 min show a clear trend of decreasing NC size over time (Figure S5, Supporting Information). Specifically, the average diameters of the NCs at each interval were measured as 14.86 ± 3.87 nm (Figure S5d, Supporting Information), 8.82 ± 1.35 nm (Figure S5e, Supporting Information), and 8.59 ± 1.21 nm (Figure S5f, Supporting Information), indicating a consistent reduction in particle size as the reaction progresses. This size reduction likely reflects the ongoing cation exchange process, which drives the structural transformation and impacts the crystal dimensions. The transformation from Cs_3_BiBr_6_ NCs to Cs_2_AgBiBr_6_ NCs results in significant changes in morphology and size, indicating that the addition of Ag^+^ and the passivation effect of water at the interface both play important roles.^[^ ^12^ ^]^ In this context, the introduction of Ag^+^ ions as dopants begins the reorganization of the Cs_3_BiBr_6_ structure, leading to the formation of Cs_2_AgBiBr_6_. Water as a medium facilitates the efficient ionization of Ag^+^ ions, simplifying their manipulation. This efficient ionization assists in the controlled transfer of ions into the toluene phase via the water‐toluene interface, enabling the controlled transformation of NCs and promoting the reaction process. As the alloying reaction proceeds, water slowly etches imperfect [BiBr_6_]^3−^ octahedra at the interface, reducing surface defects and removing possible impurities and excess ligands. The etching‐driven process led to the evolution of the previously relatively big hexagonal Cs_3_BiBr_6_ NCs into smaller and more uniform square Cs_2_AgBiBr_6_ NCs. The HR‐TEM images (Figure 1c,d) show that the Cs_3_BiBr_6_ NCs and Cs_2_AgBiBr_6_ NCs have clear lattice spacing stripes of 2.95 and 2.81 Å, corresponding to the (62‐2) plane of Cs_3_BiBr_6_ NCs and (400) plane of Cs_2_AgBiBr_6_ NCs respectively. The energy dispersive X‐ray spectroscopy in scanning transmission electron microscopy (STEM‐EDS) (Figure S6a–f, Supporting Information) element mapping images show the element distribution in which the ratio of Ag^+^ to Bi^3+^ is ≈1:1.

**Figure 2:**
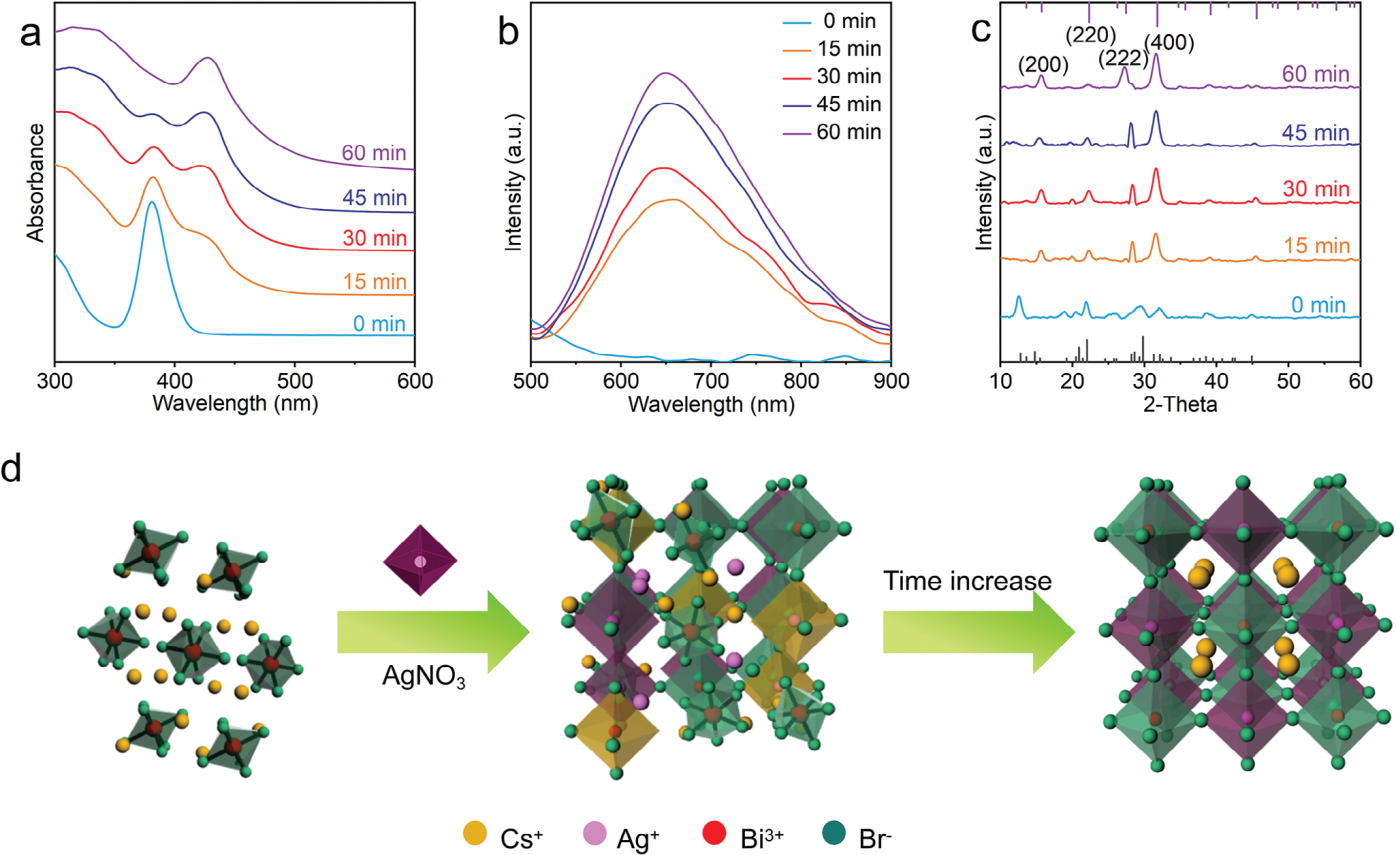
Optical and morphological properties evolution of the Cs_2_AgBiBr_6_ NCs transformation reaction from the Cs_3_BiBr_6_ NCs after adding Ag^+^ ions over time. a) Absorption spectra, b) PL spectra, c) XRD patterns, d) Schematic illustration of the Cs_2_AgBiBr_6_ synthetic process.

To clarify this composition‐structure‐property relationship, we conducted a study of the mechanism of structural evolution. The slow reaction rate of structural transformation in post‐synthetic method allows us to closely observe the transformation process in real time, making it convenient to investigate the transformation mechanism. The absorption, PL, XRD, and X‐ray photoelectron spectroscopy (XPS) were measured at intervals of 15 min to investigate the transformation process (**Figure** **2**; Figure S7, Supporting Information). The absorption spectra in Figure 2a reveal that at 15 min, a new absorption peak appears at 430 nm. As time progresses, the intensity of the absorption peak at 430 nm gradually increases, whereas the intensity of the initial Cs_3_BiBr_6_ NCs absorption peak at 380 nm gradually decreases. Eventually, the absorption peak at 380 nm disappears, leaving only the absorption peak at 430 nm. All these observations indicate that the mechanism of the alloy formation process involves a relatively slow insertion of Ag^+^ ions, which is completed at 60 min. The difference in excitonic peak energies between Cs_3_BiBr_6_ NCs and Cs_2_AgBiBr_6_ NCs can be attributed to their chemical composition and structural characteristics. The absence of Ag ions in Cs_3_BiBr_6_ and their presence in Cs_2_AgBiBr_6_ significantly alter the electronic structure. Specifically, the introduction of Ag^+^ ions in Cs_2_AgBiBr_6_ leads to a reduction in the bandgap, resulting in a lower energy excitonic peak compared to Cs_3_BiBr_6_. Additionally, the lattice structure and any associated distortions also influence the electronic band structure. Cs_3_BiB_6_ exhibits greater local structural asymmetry, which can increase the bandgap and, consequently, result in a higher energy excitonic peak. The PL spectra in Figure 2b reveal the progression of the insertion of the Ag^+^ ions process. At the start of the reaction, Cs_3_BiBr_6_ does not show any significant PL. However, after adding Ag^+^ ions at 15 min, a broad PL band at 655 nm appeared with many shoulders, which can be attributed to reaction intermediates. Subsequently, the PL band blue shifts to 650 nm and becomes more prominent over time, reaching maximum intensity at 60 min and stabilizing thereafter. The XRD patterns (Figure 2c) show a gradual transformation of the crystal structure from an initial 0D monoclinic phase to a 3D cubic DP structure. As the reaction time increased, the DP crystals diffraction pattern became more prominent. The (400) peak of Cs_2_AgBiBr_6_ shifts from 29.7° to 31.6°, stabilizing at 31.6° after 15 min. The smaller ionic radius of Ag^+^ cations (ionic radius: 1.15 Å) compared to Cs^+^ cations (ionic radius: 1.67 Å) leads to a lattice contraction, consistent with the lattice contraction observed in Figure 1c,d. Raman spectra (Figure S7a, Supporting Information) show that after the addition of Ag^+^ ions, the characteristic peak of Cs_3_BiBr_6_ NCs at 157 cm⁻¹ shifts to the characteristic peak of Cs_2_AgBiBr_6_ NCs at 187 cm⁻¹, indicating the successful transformation. The XPS spectra (Figure S7b) highlight the binding energies associated with the Ag 3d core levels. As the reaction time increases, from 0 to 60 min, the emergence of distinct peaks for Ag at binding energies ≈368 and 374 eV confirms the incorporation of Ag^+^ ions into the Cs_3_BiBr_6_ NCs. This suggests that the insertion of Ag^+^ ions facilitates the formation of the Cs_2_AgBiBr_6_ phase. The shifting and broadening of these peaks over time further illustrate the progression of the transformation process. The synthetic process of Cs_2_AgBiBr_6_ DP is shown in Figure 2d. A large number of Ag^+^ cations are added to Cs_3_BiBr_6_, where Ag^+^ ions replace Cs^+^ ions, altering the ratio between the 0D Cs_3_BiBr_6_ phase and the 3D Cs_2_AgBiBr_6_ phase. The [AgBr_6_]^5−^ and [CsBr_6_]^5−^ octahedral units are created by Br^−^ ions shared with the [BiBr_6_]^3−^ octahedra in each NC. The cubic DP crystal motif appears and coexists with the original 0D perovskite structure. Over time, Ag^+^ and Bi^3+^ fully occupied the B^+^ and B^3+^ sites of the DP crystal structure, and a pure Cs_2_AgBiBr_6_ DP is obtained. Ag^+^ and Bi^3+^ ions are located at the B^+^ and B^3+^ sites, where they combine with Br^−^ to create two distinct types of regular octahedra.^[^ ^13^ ^]^ The octahedra are interconnected in an alternating pattern and form a rock‐salt face‐centered cubic structure. Larger Cs^+^ ions fill the gaps between these octahedra, resulting in the characteristic A_2_B^+^B^3+^X_6_ structure.^[^ ^14^ ^]^ After successfully demonstrating the transformation of 0D perovskite into 3D DP, we explored the reverse transformation of 3D DP to 0D perovskite. The conversion of Cs_2_AgBiBr_6_ to Cs_3_BiBr_6_ was successfully achieved by adding tributylphosphine (TBUP) to Cs_2_AgBiBr_6_ (Figure S8, Supporting Information). It is expected that the strong binding of phosphine and Ag^+^ cations will lead to the formation of Cs_3_BiBr_6_.^[^ ^8a^ ^]^ This experiment provides a strategy for the transformation of DPs into other types of perovskites and can serve as a methodology for decomposing DPs at room temperature.

**Figure 3:**
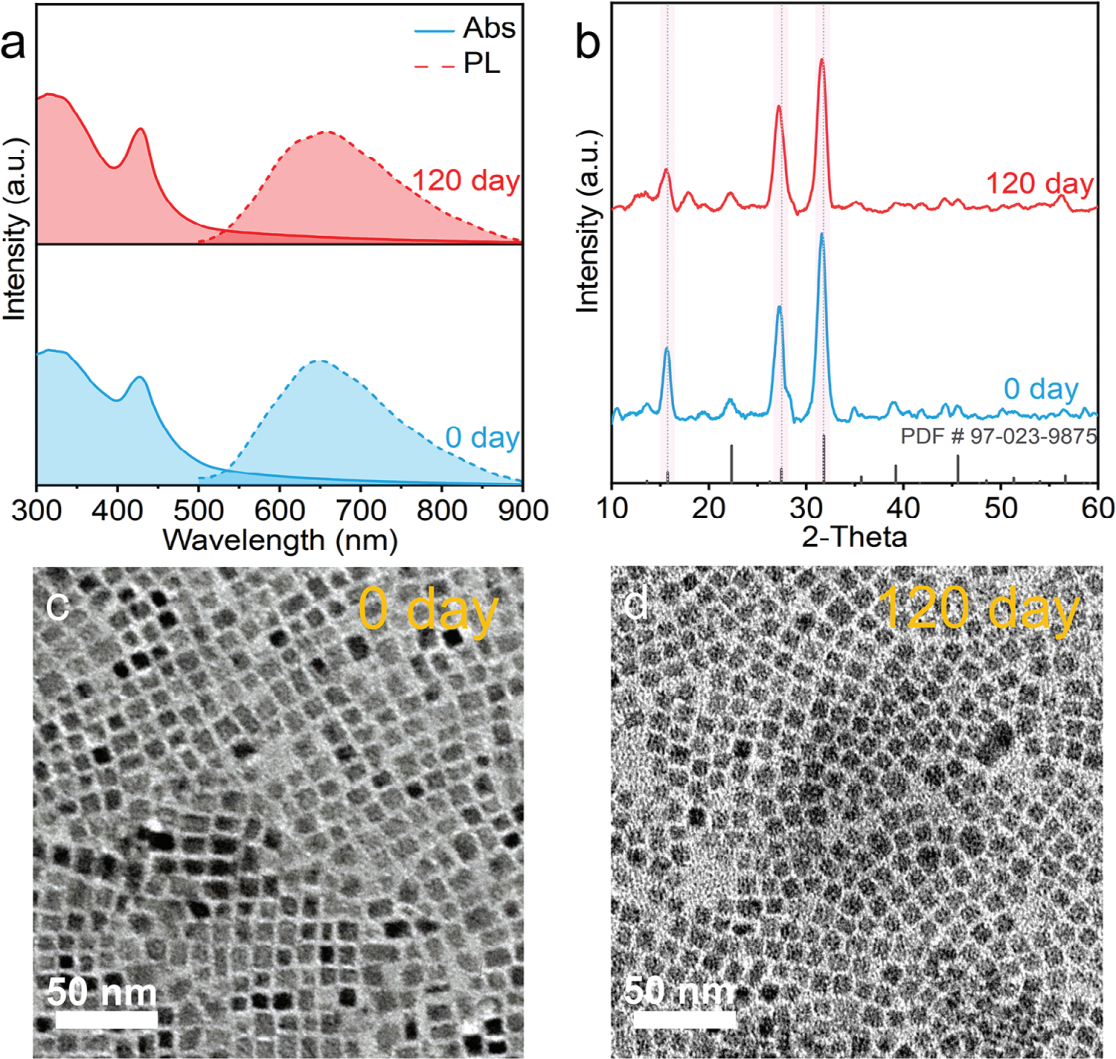
a) Absorption and PL spectra, b) XRD patterns, TEM images of Cs_2_AgBiBr_6_ for 0 day (c) and 120 days (d).

The stability of NCs is a key parameter for their potential applications. Cs_2_AgBiBr_6_ NCs synthesized by Yang et al.^[^ ^4b^ ^]^ using an acetonitrile‐hexane post‐treatment method remained stable under ambient conditions for at least 42 days, while those synthesized by Zhou et al.^[^ ^15^ ^]^ via the hot‐injection method showed stability for over 90 days at 55% relative humidity. To evaluate the stability of the Cs_2_AgBiBr_6_ NCs synthesized via the water‐oil interfacial post‐synthetic method, the properties of freshly prepared samples and those stored under ambient conditions for 120 days were compared. The absorption and PL spectra (**Figure** **3**a; Figure S9, Supporting Information) show no significant changes after 120 days, and the XRD patterns (Figure 3b) indicate that the peaks remained unchanged. Furthermore, TEM images (Figure 3c,d) reveal no notable changes in morphology. The distinct phase separation at the water‐oil interface provides a controlled environment for the growth of NCs, effectively reducing defects and enhancing structural integrity. The slow diffusion of precursors across the interface promotes the orderly integration of ions into the crystal lattice, thereby decreasing the defect density. Additionally, the aqueous phase acts as a reservoir for byproducts and impurities, effectively removing instabilities from the system, thereby further enhancing the crystal purity and stability. The controlled growth kinetics at the interface, combined with the stabilizing effects of the ligands on the surface, are key to the long‐term environmental stability of Cs_2_AgBiBr_6_ NCs. This ensures that the NCs can withstand external environmental influences and maintain their optical and structural properties stably.

**Figure 4:**
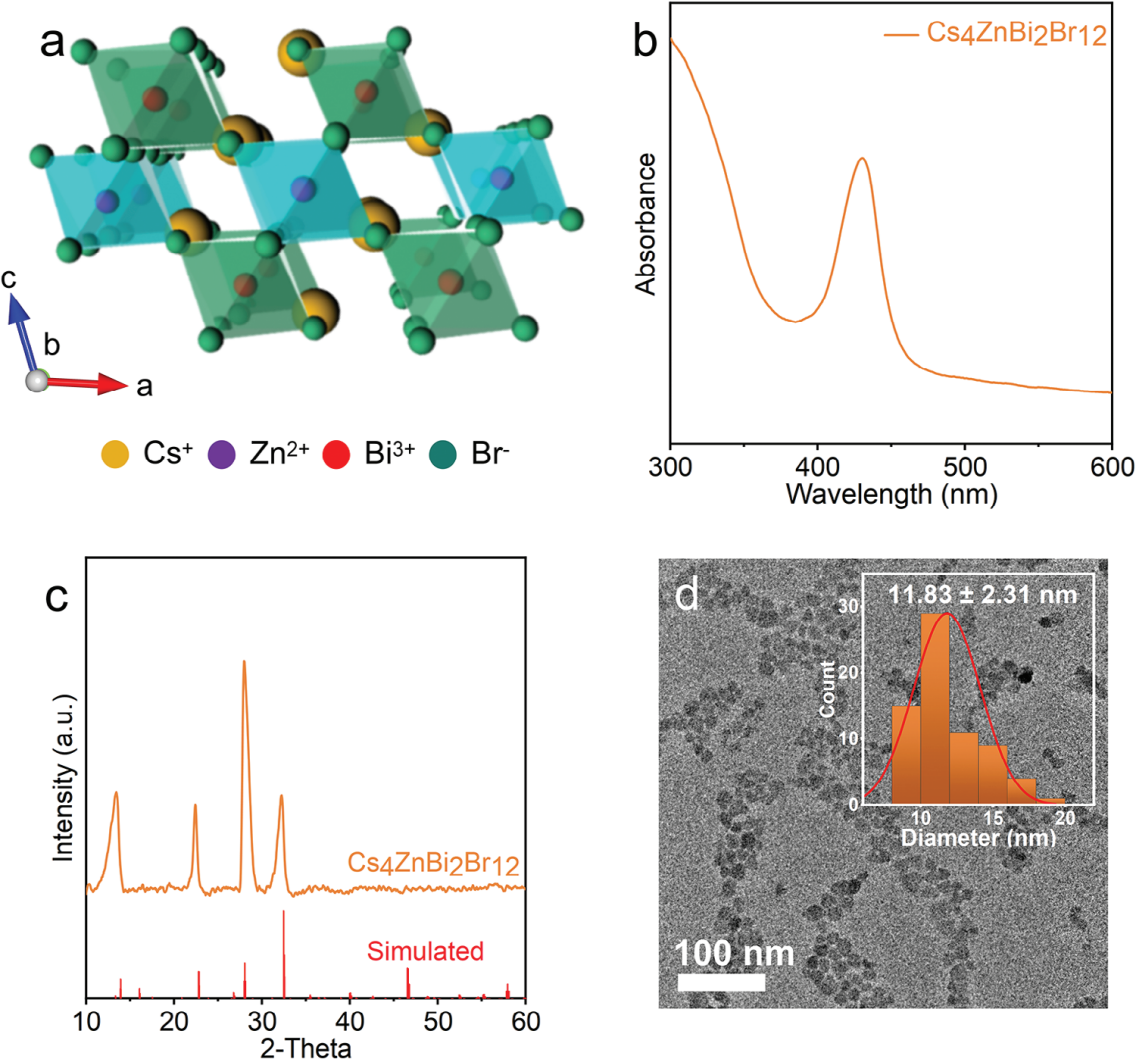
a) Structures of Cs_4_ZnBi_2_Br_12_ in bulk, b) absorption spectrum, c) XRD pattern, d) TEM image of Cs_4_ZnBi_2_Br_12_ NCs and particle size distribution histograms (inset).

To extend the water‐oil interfacial post‐synthetic method for synthesizing perovskite NCs to other types, we also synthesized 2D LDP Cs_4_ZnBi_2_Br_12,_ which is one of the most promising p‐type transparent conductor materials.^[^ ^16^ ^]^ The structure of Cs_4_ZnBi_2_Br_12_ is shown in **Figure** **4**a. Bi^3+^ cations and Zn^2+^ cations are coordinated with six Br^−^ ions, forming two types of octahedral blocking units. Specifically, the [ZnBr_6_]^4−^ octahedral layer is sandwiched between two [BiBr_6_]^3−^ layers via corner‐sharing to form a [BiBr_6_]^3−^‐[ZnBr_6_]^4−^‐[BiBr_6_]^3−^ triple‐layered perovskite structure. The Cs^+^ cation was used to fill the gaps and strike a charge balance.^[^ ^17^ ^]^ In this case, a certain amount of ZnBr_2_ aqueous solution is added to the Cs_3_BiBr_6_ precursor and stirred for 1 h to fully react (more details are given in Support Information). The absorption spectrum of Cs_4_ZnBi_2_Br_12_ shows a distinct peak at 430 nm (Figure 4b), whereas PL is not detected at room temperature. The XRD pattern shown in Figure 4c displays peaks at 22.7°, 26.9°, and 32.3°, which match well with the simulated XRD patterns (Table S2, Supporting Information). Figure 4d shows the TEM image of Cs_4_ZnBi_2_Br_12_ NCs, which possess sphere‐like shape. Compared with the hexagon presented at the beginning of Cs_3_BiBr_6_, the shape is changed, and it indicates that there is a large variation in the crystal structure of NCs, which is the result of the interaction of water and Zn^2+^. Thus, these characterizations indicate the successful synthesis of Cs_4_ZnBi_2_Br_12_.

The transformation process of Cs_3_BiBr_6_ NCs to Cs_4_ZnBi_2_Br_12_ NCs was systematically studied by using absorption spectra, XRD, Raman spectroscopy, XPS, and TEM at 10‐min intervals (Figures S10 and S11, Supporting Information). This real‐time transformation analysis provides insights into the dynamic structural and compositional changes during the cation insertion process. Figure S10a (Supporting Information) illustrates the evolution of the absorption spectra over time, showing progressive red shifts in the absorption peaks as Zn^2+^ ions are inserted into the Cs_3_BiBr_6_. This shift indicates gradual changes in the electronic structure, which can be attributed to the structural reorganization associated with the 0D to 2D transformation. The XRD patterns (Figure S10b, Supporting Information) reveal a clear phase transition, with new diffraction peaks emerging at 20 min, indicating the formation of Cs_4_ZnBi_2_Br_12_ crystallites. The peaks become defined and intense over the reaction period, indicating that the transformation largely completes around the 30‐min mark. This phase evolution strongly suggests a steady incorporation of Zn^2+^ ions, leading to the formation of the layered perovskite structure. Raman spectra (Figure S10c, Supporting Information) further substantiate the structural transformation, with distinct vibrational modes appearing as Zn^2+^ incorporation progresses. These newly emerging peaks reflect the development of the LDP structure, characterized by enhanced structural order and distinct bonding environments compared to the initial Cs_3_BiBr_6_ phase. XPS analysis (Figure S10d, Supporting Information) of the Zn 2p region confirms the presence and gradual increase of Zn content within the NCs. At 10 min, only a small Zn signal is detected, while the Zn signal at 30 min is notably stronger, indicating substantial Zn^2+^ incorporation. This gradual increase supports the hypothesis that the transformation is driven by a slow cation insertion mechanism. TEM images (Figure S11a,b, Supporting Information) and particle size distributions (Figure S11c,d, Supporting Information) corroborate the structural reorganization from Cs_3_BiBr_6_ NCs to Cs_4_ZnBi_2_Br_12_ NCs. TEM images reveal that the particle diameter decreases from 17.75 ± 3.61 nm at 10 min to 13.44 ± 2.08 nm at 20 min, and further to 11.83 ± 2.31 nm at 30 min (Figure 4d; Figure S11c,d, Supporting Information), suggesting that the Zn^2+^ insertion facilitates the restructuring and slight compaction of the crystal lattice. This transformation reflects the particle‐size reduction induced by water‐driven etching, resulting in a distinct morphology and particle‐size distribution compared to the initial 0D Cs_3_BiBr_6_ NCs. These observations collectively reveal a transformation mechanism for Cs_4_ZnBi_2_Br_12_ NCs that aligns closely with the cation‐insertion pathway observed in Cs_2_AgBiBr_6_. We have further extended this method to synthesize Cs_4_CdBi_2_Cl_12_ (Figure S12, Supporting Information), thereby reaffirming the universality of method. Figure S12a (Supporting Information) shows the absorbance spectrum characterized by a distinct peak at ≈380 nm. Figure S12b (Supporting Information) presents the XRD pattern of Cs_4_CdBi_2_Cl_12_ the congruence between the experimental XRD pattern and the simulated data (Table S3, Supporting Information) confirms the successful synthesis of the targeted materials. This universality encompasses the use of Cl^−^ ions and various other metal ions, underscoring the adaptability of this approach to a broad range of compositions.

## 3 Conclusion

We present a reliable and efficient water‐oil biphasic interface‐driven method for synthesizing 3D DP Cs_2_AgBiBr_6_ NCs and 2D LDP Cs_4_ZnBi_2_Br_12_ NCs. The Cs_2_AgBiBr_6_ NCs produced via this approach exhibited uniform particle size, high crystallinity, and remarkable structural stability, retaining their integrity for over 120 days under ambient conditions. Through a detailed investigation of the optical properties and structural evolution of Cs_2_AgBiBr_6_ NCs and Cs_4_ZnBi_2_Br_12_ NCs, we revealed the critical role of slow cation insertion, driven by water‐oil interfacial processes, in facilitating structural transformation. Our findings provide a universal pathway for synthesizing high‐quality DP NCs and offer valuable insights into the relationship between structural dynamics and material properties.

## Conflict of Interest

The authors declare no conflict of interest.

## Supporting information



Supporting Information

## Data Availability

The data that support the findings of this study are available from the corresponding author upon reasonable request.

## References

[1] a) M. M. Liu , Q. Wan , H. M. Wang , F. Carulli , X. C. Sun , W. L. Zheng , L. Kong , Q. Zhang , C. Y. Zhang , Q. G. Zhang , S. Brovelli , L. Li , Nat. Photonics 2021, 15, 379;

[2] a) Y. Liu , X. M. Rong , M. Z. Li , M. S. Molokeev , J. Zhao , Z. G. Xia , Angew. Chem., Int. Ed. 2020, 59, 11634; 10.1002/anie.202004562 32329132

[3] a) Y. Liu , A. Nag , L. Manna , Z. G. Xia , Angew. Chem., Int. Ed. 2021, 60, 11592; 10.1002/anie.202011833 33084115

[4] a) T. Cai , W. W. Shi , S. Hwang , K. Kobbekaduwa , Y. Nagaoka , H. J. Yang , K. Hills‐Kimball , H. Zhu , J. Y. Wang , Z. G. Wang , Y. Z. Liu , D. Su , J. B. Gao , O. Chen , J. Am. Chem. Soc. 2020, 142, 11927; 32510205 10.1021/jacs.0c04919

[5] J. J. Luo , X. M. Wang , S. R. Li , J. Liu , Y. M. Guo , G. D. Niu , L. Yao , Y. H. Fu , L. Gao , Q. S. Dong , C. Y. Zhao , M. Y. Leng , F. S. Ma , W. X. Liang , L. D. Wang , S. Y. Jin , J. B. Han , L. J. Zhang , J. Etheridge , J. B. Wang , Y. F. Yan , E. H. Sargent , J. Tang , Nature 2018, 563, 541. 30405238 10.1038/s41586-018-0691-0

[6] a) W. C. Pan , H. D. Wu , J. J. Luo , Z. Z. Deng , C. Ge , C. Chen , X. W. Jiang , W. J. Yin , G. D. Niu , L. J. Zhu , L. X. Yin , Y. Zhou , Q. G. Xie , X. X. Ke , M. L. Sui , J. Tang , Nat. Photonics 2017, 11, 726;

[7] E. Greul , M. L. Petrus , A. Binek , P. Docampo , T. Bein , J. Mater. Chem. A 2017, 5, 19972.

[8] a) S. E. Creutz , E. N. Crites , M. C. De Siena , D. R. Gamelin , Nano Lett. 2018, 18, 1118; 29376378 10.1021/acs.nanolett.7b04659

[9] a) Y. Bekenstein , J. C. Dahl , J. Huang , W. T. Osowiecki , J. K. Swabeck , E. M. Chan , P. Yang , A. P. Alivisatos , Nano Lett. 2018, 18, 3502; 29719146 10.1021/acs.nanolett.8b00560

[10] L. Z. Wu , Y. Wang , M. Kurashvili , A. Dey , M. H. Cao , M. Döblinger , Q. Zhang , J. Feldmann , H. Huang , T. Debnath , Angew. Chem., Int. Ed. 2022, 61, e202115852. 10.1002/anie.202115852 PMC9305410 34995399

[11] a) J. Yin , P. Maity , M. De Bastiani , I. Dursun , O. M. Bakr , J. L. Brédas , O. F. Mohammed , Sci. Adv. 2017, 3, e1701793; 29250600 10.1126/sciadv.1701793 PMC5731998

[12] a) C. H. Bi , Z. W. Yao , X. J. Sun , X. C. Wei , J. X. Wang , J. J. Tian , Adv. Mater. 2021, 33, 2006722; 10.1002/adma.202006722 33629762

[13] a) E. Hofman , A. Khammang , J. T. Wright , Z. J. Li , P. F. McLaughlin , A. H. Davis , J. M. Franck , A. Chakraborty , R. W. Meulenberg , W. W. Zheng , J. Phys. Chem. Lett. 2020, 11, 5992; 32633980 10.1021/acs.jpclett.0c01861

[14] a) X. Q. Yang , W. Wang , R. Ran , W. Zhou , Z. P. Shao , Energy Fuels 2020, 34, 10513;

[15] L. Zhou , Y. F. Xu , B. X. Chen , D. B. Kuang , C. Y. Su , Small 2018, 14, e1703762. 29380522 10.1002/smll.201703762

[16] J. Xu , J. B. Liu , J. F. Wang , B. X. Liu , B. Huang , Adv. Funct. Mater. 2018, 28, 1800332.

[17] J. H. Wei , J. F. Liao , X. D. Wang , L. Zhou , Y. Jiang , D. B. Kuang , Matter 2020, 3, 892.

