# Supplementary material for: Interfacial Structural Transformation for the Synthesis of Lead‐Free Double Perovskite Nanocrystals
Source: Adv Sci (Weinh). 2025 Mar 8;12(17):2416046. doi: 10.1002/advs.202416046 (PMC12061304; doi:10.1002/advs.202416046)
Supplement: Supplementary file 1 — Supporting Information [file ADVS-12-2416046-s001.docx]

**Interfacial Structural Transformation for the Synthesis of Lead-Free Double Perovskite Nanocrystals in Water-Oil Biphase**

Jun Liu,^[a],[b]^ Anna A Vedernikova,^[c]^ Qi Xue,*^[d]^ Huiying Gao, ^[d]^ Xiuhui Xie,^[a],[b]^ Jinfeng Xie,^[a],[b]^ Elena V Ushakova,^[c],[e]^ He Huang,*^[a],[b]^ Xiaohong Zhang*^[f]^

[a] Jun Liu, Xiuhui Xie, Jinfeng Xie, Prof. Dr. H. Huang, School of Optoelectronic Science and Engineering &, Collaborative Innovation Center of Suzhou Nano Science and Technology, Soochow University, 215006, Suzhou, China. E-mail: hh@suda.edu.cn.

[b] Jun Liu, Xiuhui Xie, Jinfeng Xie, Prof. Dr. H. Huang, Key Lab of Advanced Optical Manufacturing Technologies of Jiangsu Province &, Key Lab of Modern Optical Technologies of Education Ministry of China, Soochow University, 215006, Suzhou, China.

[c] Anna A Vedernikova, Dr. Elena V Ushakova, International Research and Education Centre for Physics of Nanostructures, ITMO University, Saint Petersburg 197101, Russia.

[d] Dr. Qi Xue, Huiying Gao, School of Physical Science and Technology, Soochow University, 215006, Suzhou, China. E-mail: xq@suda.edu.cn.

[e] Dr. Elena V Ushakova, Department of Materials Science and Engineering, and Centre for Functional Photonics (CFP), City University of Hong Kong, Hong Kong SAR 999077, P. R. China.

[f] Prof. Dr. Xiaohong Zhang, Institute of Functional Nano and Soft Materials (FUNSOM) Jiangsu Key Laboratory for Carbon-Based Functional Materials and Devices Soochow University Suzhou 215123, P. R. China. E-mail: xiaohong_zhang@suda.edu.cn.

Materials

All the chemicals were used as received without further purification: oleylamine (70%, Sigma-Aldrich), oleic acid (90%, Sigma-Aldrich), octadecene (90%, Sigma-Aldrich), cesium carbonate (Cs_2_CO_3_, 99.9%, Aladdin), bismuth acetate (Bi(OAc)_3_, >99.99%, Aladdin), bromotrimethylsilane (TMS-Br, 97%, Aladdin), trimethylchlorosilane (TMS-Cl, 99%, Aladdin), Cadmium nitrate (CdNO_2_, 99%, Aladdin), silver nitrate (AgNO_3_,>99%, Aladdin), zinc bromide (ZnBr_2_, 98%, Aladdin)， tributylphosphine (TBUP, 95%, Aladdin ) Toluene and ethyl acetate were obtained from Sinopharm Chemical Reagent Co., Ltd, China.

Synthesis of Cs_3_BiX_6_ (X= Cl, Br) nanocrystals (NCs)

Cesium carbonate (42 mg, 0.13 mmol), bismuth(III) acetate (97 mg, 0.26 mmol), oleylamine (1.5 mL), octadecene (10 mL), and oleic acid (0.5 mL) were combined in a 50 mL three-neck round-bottom flask. The mixture was degassed and dried by stirring and heating to 120 ℃ under vacuum for 1 h. Then, under an N_2_ atmosphere, the reaction mixture was heated to 180 ℃, and 500 μL of neat TMS-X was swiftly injected; the reaction mixture immediately became cloudy and yellow. After 15 s, the flask was immersed in an ice-water bath and cooled to room temperature. The resulting NCs were separated from the solution by centrifuge at 6000 rpm for 30 min; the supernatant was then thoroughly drained from the precipitate. The precipitate was resuspended in 10 mL toluene and centrifuged again for 20 min. Then purified twice with toluene and ethyl acetate. The resulting NCs were dispersed in toluene for further use.

Post-synthetic transformation of Cs_3_BiBr_6_ NCs to double perovskite (DP) Cs_2_AgBiBr_6_ NCs or layered double perovskite (LDP) Cs_4_ZnBi_2_Br_12_ NCs

In a typical experiment, certain amounts of 2g/mL AgNO_3_ solution in water were added to as-prepared Cs_3_BiBr_6_ NCs, specifically 4.4 μL of the AgNO₃ solution was introduced into 2 mL of the prepared NCs dispersion. Subsequently, the two-phase mixed solution was stirred at the speed of 400 rpm for 1 h at ambient conditions. Then, after a minute of phase separation, the toluene was pipetted out from the upper phase. The toluene phase was then transferred to a centrifuge tube and treated by centrifugation at the speed of 4000 rpm for 5 minutes. The precipitate was discarded, and the final product, Cs_2_AgBiBr_6_ NCs, was dispersed in toluene. For the synthesis of Cs_4_ZnBi_2_Br_12_, certain amounts of ZnBr_2_ aqueous solution (4g/mL) were added to as-prepared Cs_3_BiBr_6_ NCs, specifically 1.5 μL of the ZnBr₂ solution was introduced into 2 mL of the prepared NC dispersion. Subsequently, the two-phase mixed solution was stirred at the speed of 400 rpm for 0.5 h at ambient conditions. The other steps were the same as the front part.

Post-synthetic transformation of Cs_3_BiCl_6_ NCs to DP Cs_2_AgBiCl_6_ NCs or LDP Cs_4_CdBi_2_Cl_12_ NCs

In a typical experiment, certain amounts of 2g/mL AgNO_3_ solution in water were added to as-prepared Cs_3_BiCl_6_ NCs, specifically 4.4 μL of the AgNO₃ solution was introduced into 2 mL of the prepared NCs dispersion. Subsequently, the two-phase mixed solution was stirred at the speed of 400 rpm for 1 h at ambient conditions. Then, after a minute of phase separation, the toluene was pipetted out from the upper phase. The toluene phase was then transferred to a centrifuge tube and treated by centrifugation at the speed of 4000 rpm for 5 minutes. The precipitate was discarded, and the final product, Cs_2_AgBiCl_6_ NCs, was dispersed in toluene. For the synthesis of Cs_4_CdBi_2_Cl_12_, certain amounts of CdNO_2_ aqueous solution (1.5g/mL) were added to as-prepared Cs_3_BiCl_6_ NCs, specifically 4 μL of the CdNO_2_ solution was introduced into 2 mL of the prepared NC dispersion. Subsequently, the two-phase mixed solution was stirred at the speed of 400 rpm for 0.5 h at ambient conditions. The other steps were the same as the front part. Then, after a minute of phase separation, the toluene was pipetted out from the upper phase. The toluene phase was then transferred to a centrifuge tube and treated by centrifugation at the speed of 4000 rpm for 5 minutes. The precipitate was discarded, and the final product, Cs_4_CdBi_2_Cl_12_, was dispersed in toluene.

Post-synthetic transformation of Cs_2_AgBiBr_6_ NCs to Cs_3_BiBr_6_ NCs

In a typical experiment, 5 μL TBUP was added to 2 mL as-prepared Cs_2_AgBiBr_6_ NCs. Subsequently, the mixed solution was stirred at the speed of 400 rpm for 1 h at ambient conditions. The resulting solution was treated by centrifugation at the speed of 4000 rpm for 5 minutes. The precipitate was discarded, and the final product Cs_3_BiBr_6_ NCs dispersed in toluene.

Characterization

Transmission electron microscopy (TEM) images, HAADF-STEM, and corresponding elemental mapping images were obtained by field emission high-resolution transmission electron microscopy (FEI Talos F200X, Thermo Fisher) at 200 kV. Powder The X-ray diffraction (XRD) patterns were obtained on a desktop diffractometer (D2 PHASER, Bruker, Germany) with a Cu Kα source (λ = 1.54056 Å). The absorption spectra were detected under the illumination of the light source (Oceaninsight, DH-2000-BAL), the photoluminescence (PL) measurements were detected under 365 nm excitation (Oceaninsight, L365A), and all the data were recorded by a fluorescence spectrophotometer (Oceaninsight QEPRO, America). Raman spectra were measured using a Witec Alpha 300 confocal Raman microscope excited by a 532 nm laser. The X-ray photoelectron spectroscopy (XPS) spectra were performed on a Thermo Scientific K-Alpha instrument operating on Al Kα=1486.6 eV radiation with a spot size of ~ 200 μm.

Statistical Analysis

The size distribution of the samples was measured manually using ImageJ software. For each sample, a sufficient number of measurements, ranging from 30 to 200 representative data points, were taken to ensure the accuracy and representativeness of the data. The size distributions were then presented as histograms, with a fitted normal distribution curve overlaid to visually assess the alignment of the data with a normal distribution. This was implemented using Origin 2021. The data were expressed as the mean ± standard deviation (SD) for each sample.

Table S1. The lattice parameters for Cs_3_BiBr_6_ NCs.（CIF data of a monoclinic Cs_3_BiCl_6_ phase (ICSD# 201982) was used as the starting structure）

| Compound | Cs_3_BiBr_6_ |
| --- | --- |
| Crystal system | Monoclinic |
| Space group | C2/c |
| a (Å) | 28.31 |
| b (Å) | 8.617 |
| c (Å) | 13.71 |
| β (° ) | 99.48 |

Table S2. The lattice parameters for Cs_4_ZnBi_2_Br_12_ NCs.（CIF data of a monoclinic Cs_4_CuSbCl_6_ phase (ICSD# 243918) was used as the starting structure）

| Compound | Cs_4_ZnBi_2_Br_12_ |
| --- | --- |
| Crystal system | Monoclinic |
| Space group | C2/m |
| a (Å) | 13.79751 |
| b (Å) | 7.79919 |
| c (Å) | 13.53207 |
| β (° ) | 109.3862 |

Table S3. The lattice parameters for Cs_4_CdBi_2_Cl_12_ NCs.（CIF data of a monoclinic Cs_4_CuSbCl_6_ phase (ICSD# 243918) was used as the starting structure）

| Compound | Cs_4_CdBi_2_Cl_12_ |
| --- | --- |
| Crystal system | Monoclinic |
| Space group | C2/m |
| a (Å) | 13.06009 |
| b (Å) | 7.55175 |
| c (Å) | 13.10649 |
| β (° ) | 109.3938 |


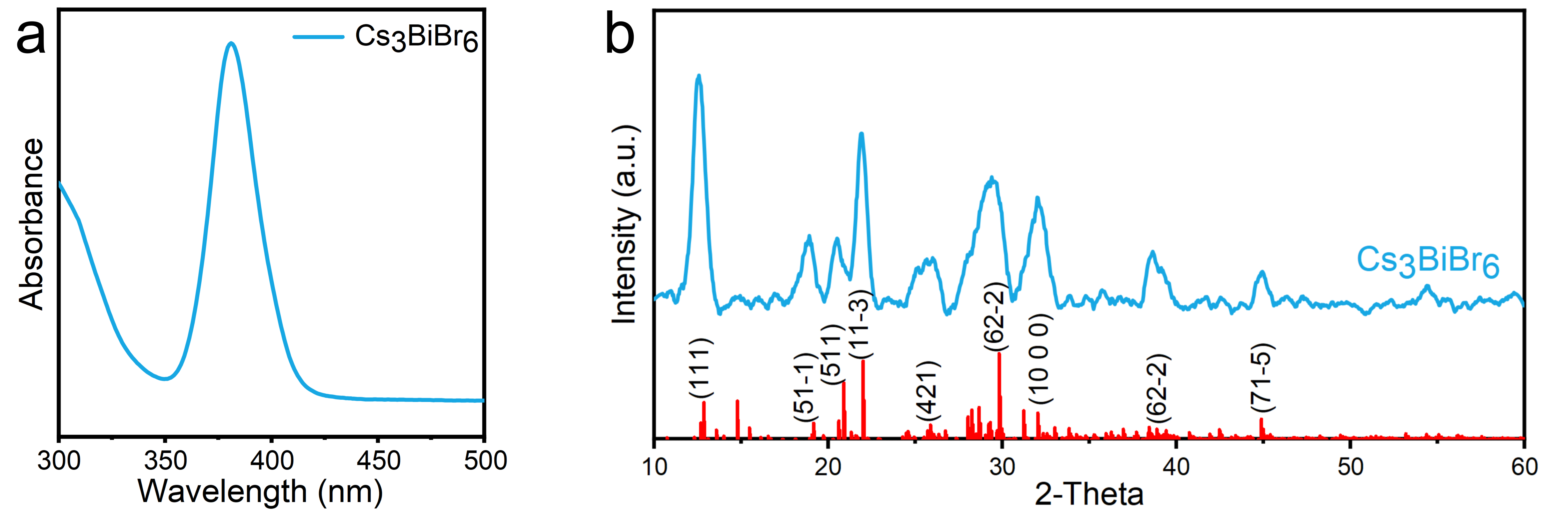


Figure S1. (a) Absorption spectrum, (b) XRD pattern of Cs_3_BiBr_6_ NCs.


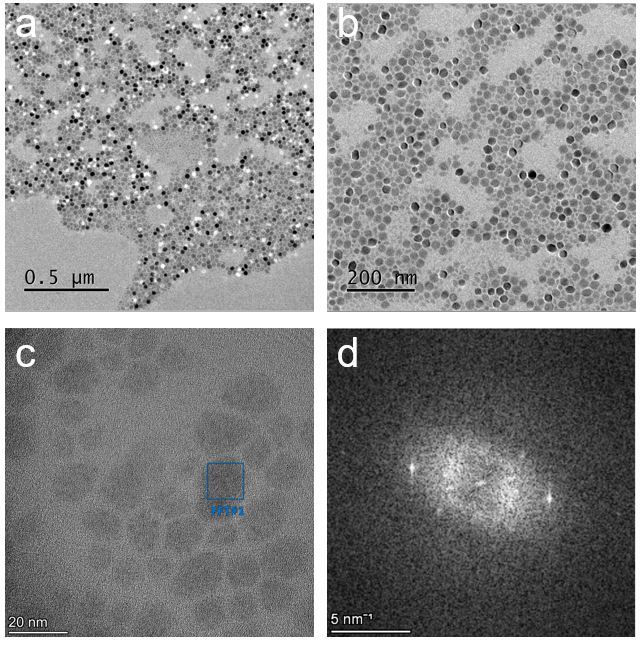


Figure S2. (a-c) TEM images, (d) FFT image of Cs_3_BiBr_6_ NCs.


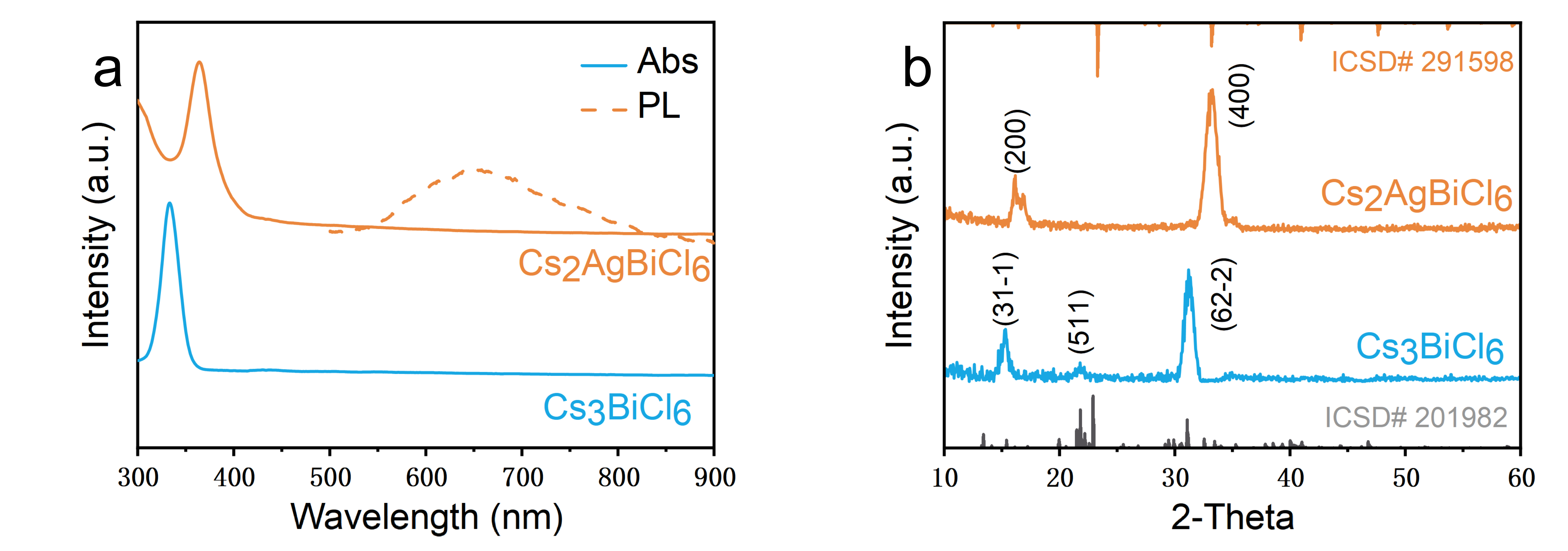


Figure S3. (a) Absorption spectra (solid lines) and PL spectra (dashed line), (b) XRD pattern of Cs_3_BiCl_6_ NCs (ICSD# 201982) and Cs_2_AgBiCl_6_ NCs (ICSD# 291598) obtained by the transformation reactions.


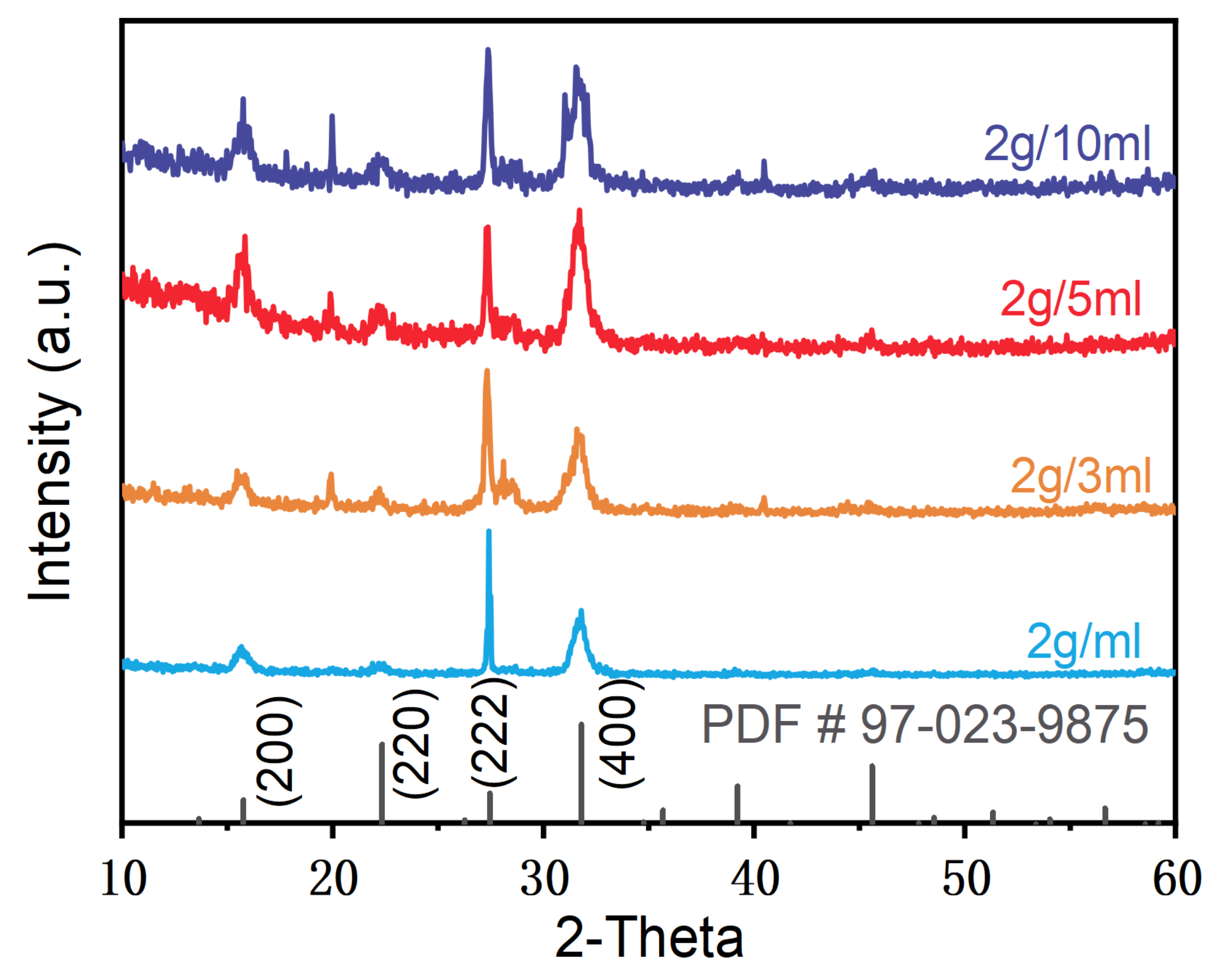


Figure S4. XRD patterns of Cs_2_AgBiBr_6_ samples synthesized with varying concentrations of AgNO₃ in the aqueous phase: 2 g/mL (11.77 M), 2 g/3 mL (3.92 M), 2 g/5 mL (2.35 M), and 2 g/10 mL (1.18 M).


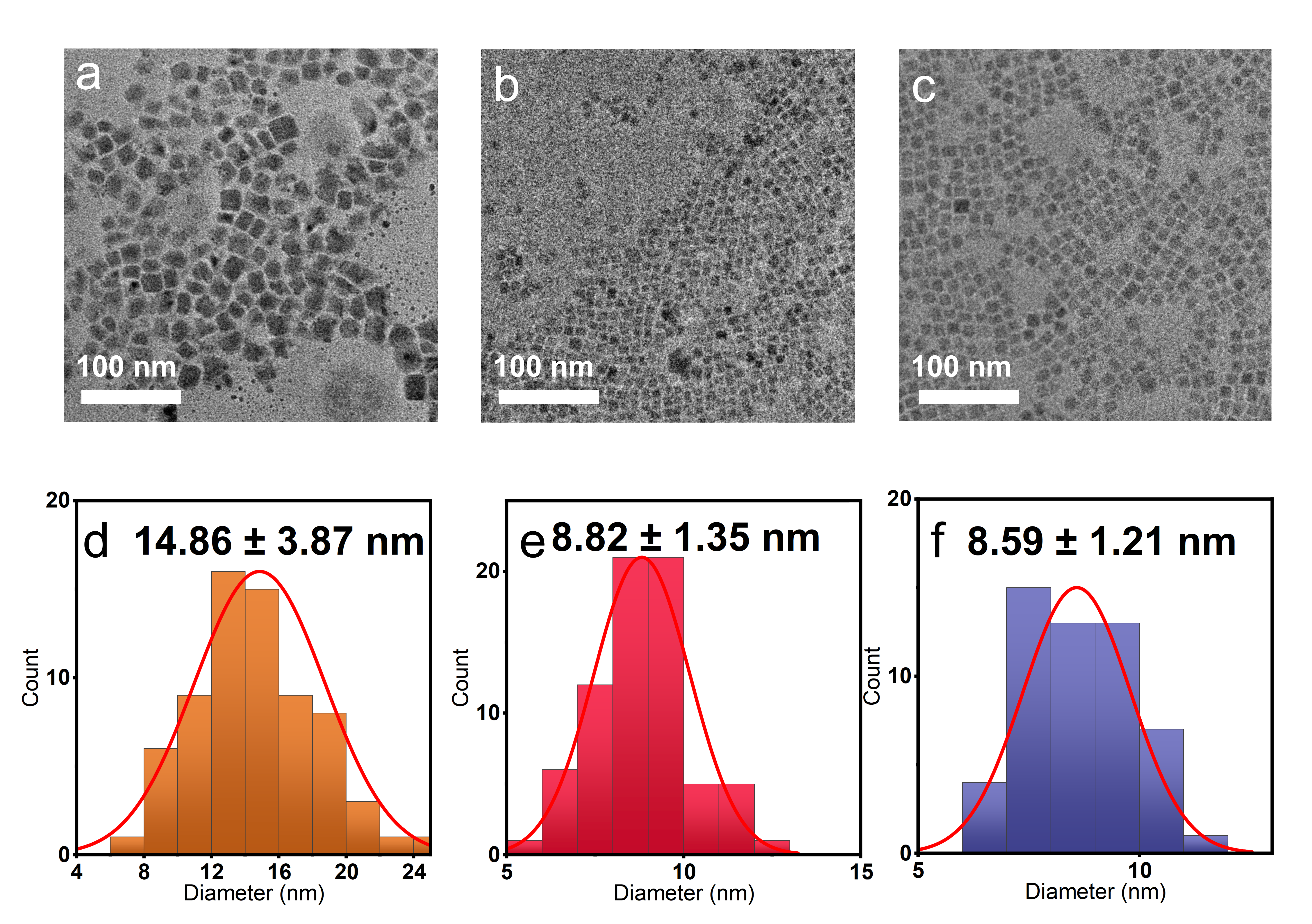


Figure S5. (a-c) TEM images, (d-f) particle size distribution histograms of Cs_3_BiBr_6_ NCs transfer to Cs_2_AgBiBr_6_ NCs (15 min, 30 min, 45 min, respectively).


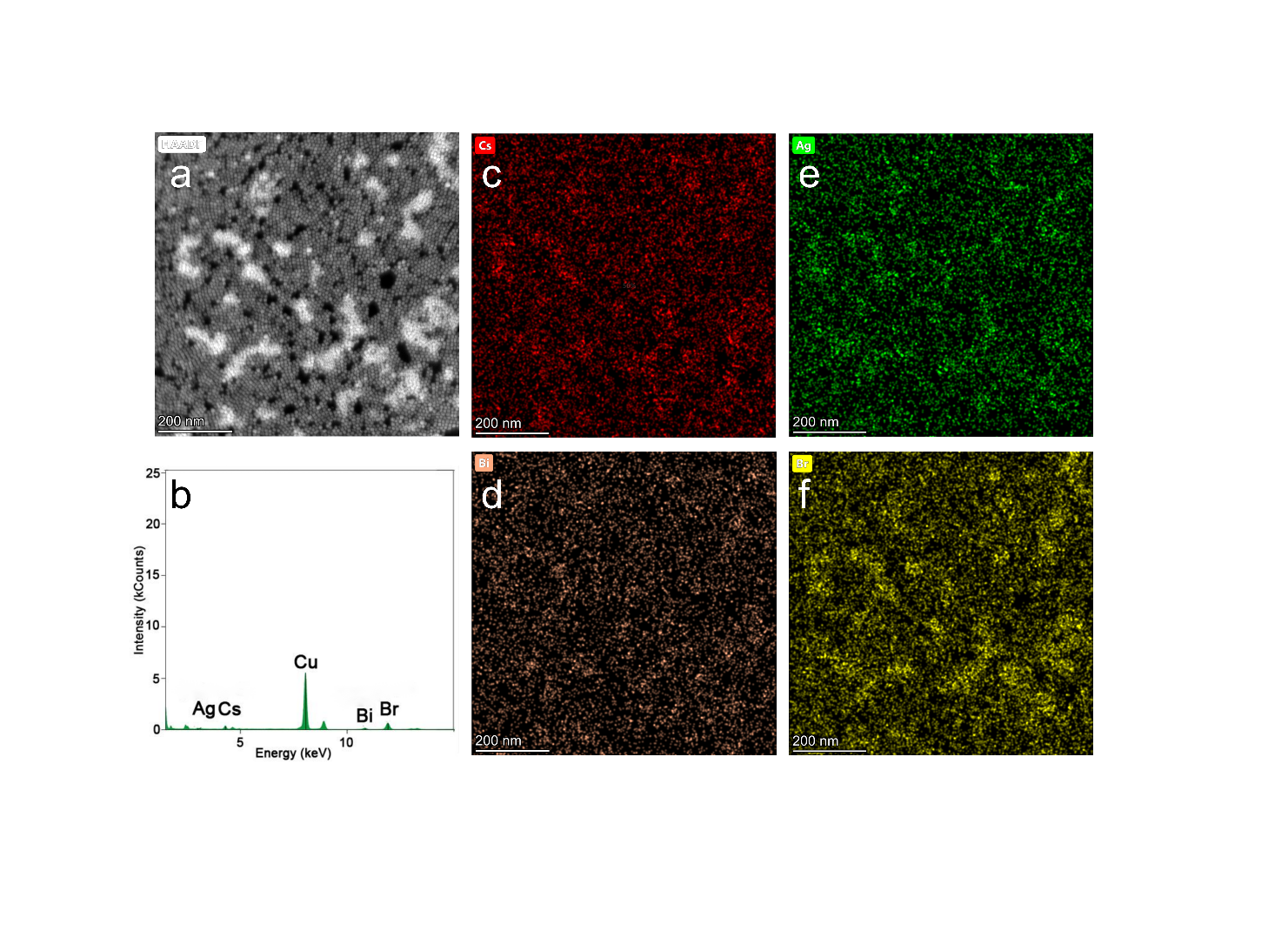


Figure S6. (a) HAADF-STEM images, (b) EDX spectra, (c-f) STEM-EDS mapping for Cs(c), Bi (d), Ag (e), Br (f) of Cs_2_AgBiBr_6_ NCs.


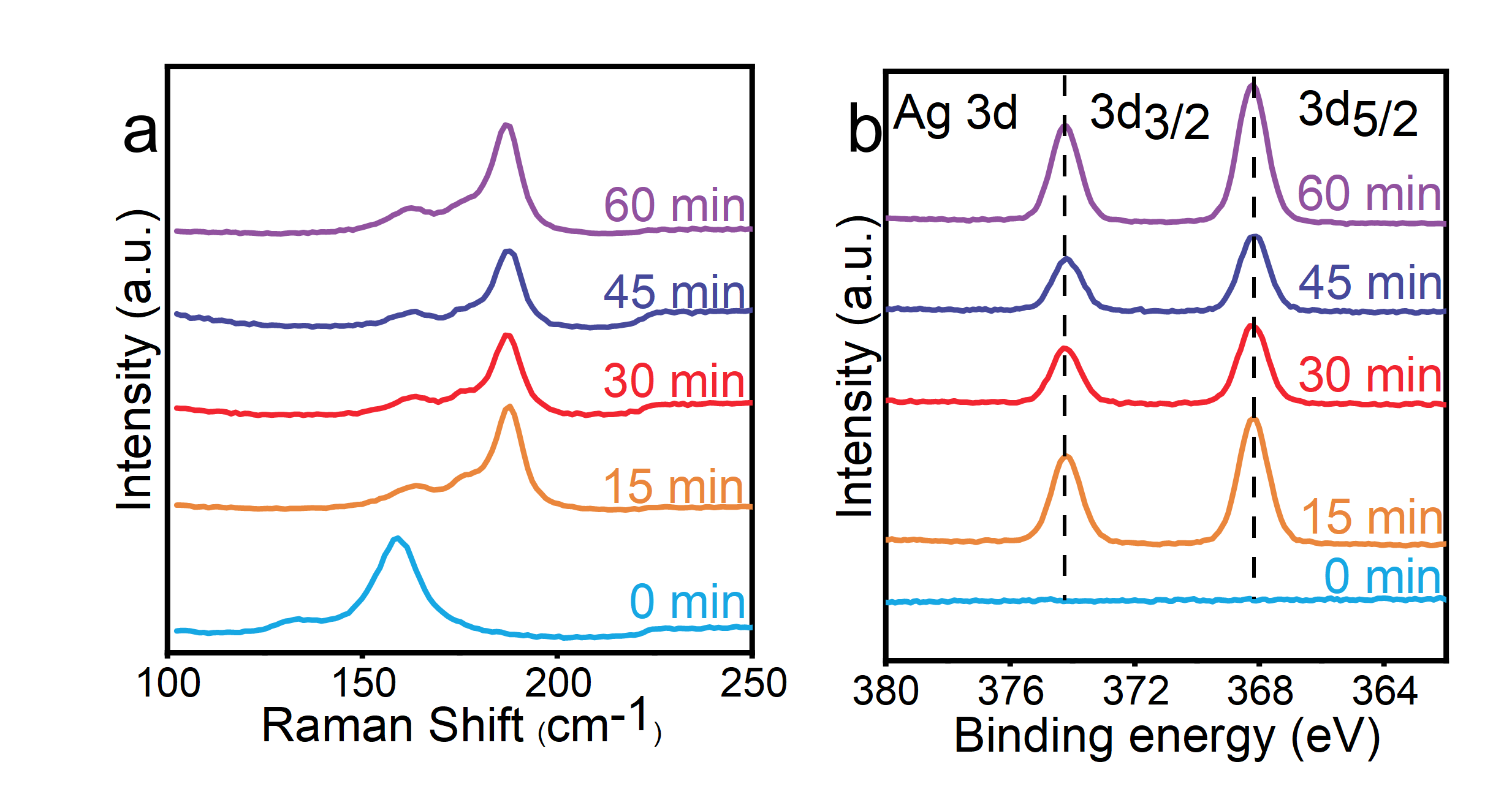


Figure S7. Morphology properties evolution of the Cs_2_AgBiBr_6_ NCs transformation reaction from the Cs_3_BiBr_6_ NCs after adding Ag^+^ ions over time. (a) Raman spectra, (b) XPS spectra of Ag.


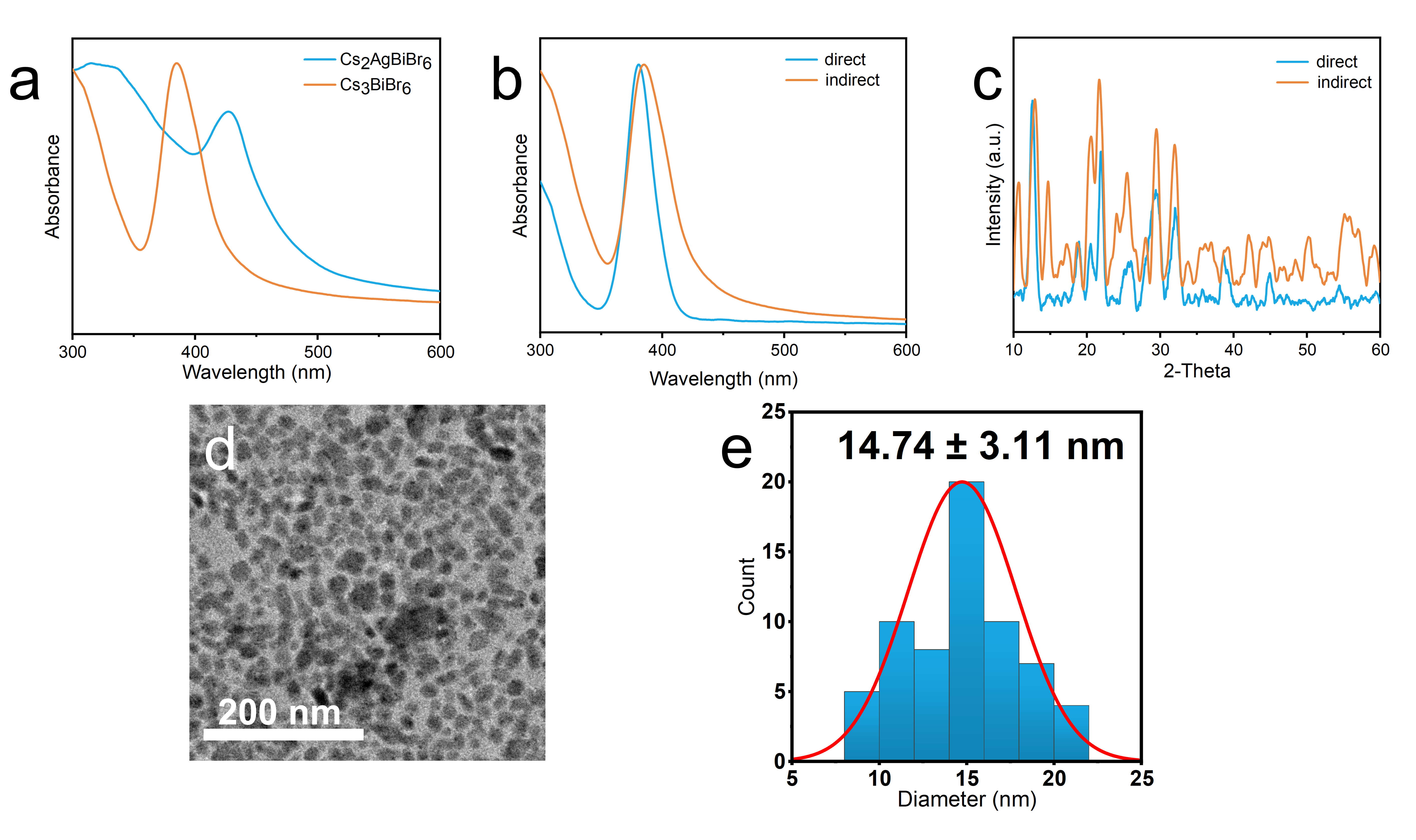


Figure S8. (a) Absorption spectra of Cs_2_AgBiBr_6_ obtained from the treatment of Cs_3_BiBr_6_ with AgNO_3_ aqueous solution (blue) and Cs_3_BiBr_6_ obtained from the treatment of Cs_2_AgBiBr_6_ with TBUP (orange) (b) PL spectra (c) XRD patterns obtained from the treatment of Cs_2_AgBiBr_6_ nanocrystals with TBUP (orange) and directly synthesized Cs_3_BiBr_6_ (blue) (d) TEM image of Cs_3_BiBr_6_ obtained from the treatment of Cs_2_AgBiBr_6_ with TBUP e (e) particle size distribution histograms Cs_3_BiBr_6_ obtained from the treatment of Cs_2_AgBiBr_6_ with TBUP D_avg_=18.15 ± 6.35 nm.


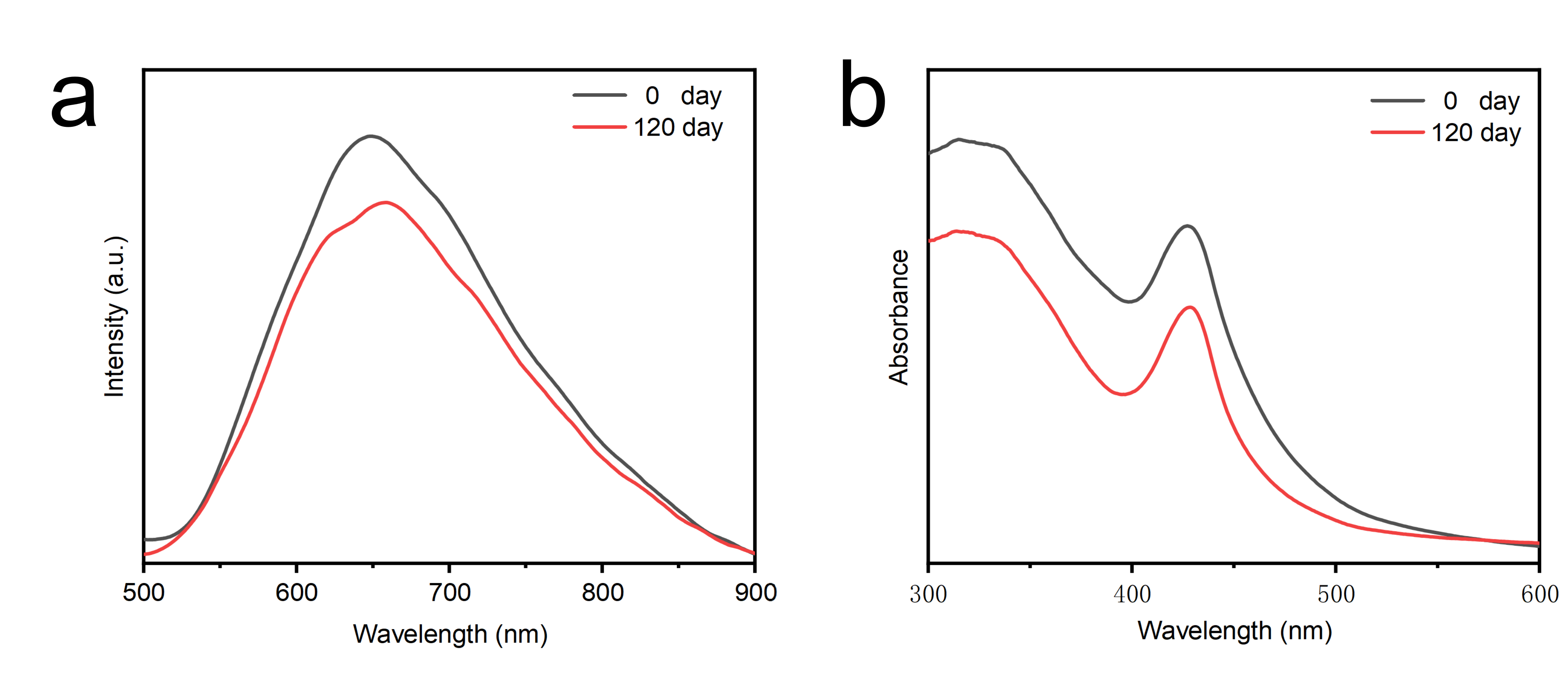


Figure S9. Cs_2_AgBiBr_6_ for 0 and 120 days (a) PL spectra (b) Absorption spectra.


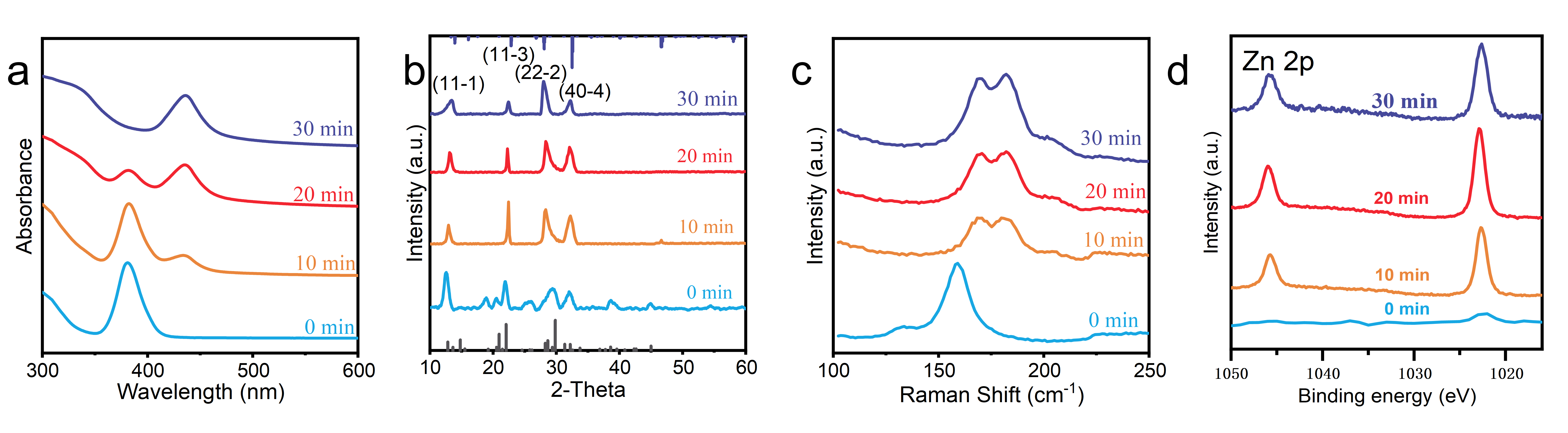


Figure S10. Optical and morphology properties evolution of the Cs_4_ZnBi_2_Br_12_ NCs transformation reaction from the Cs_3_BiBr_6_ NCs after adding Zn^2+^ ions over time. (a) Absorption spectra, (b) XRD patterns, (c) Raman spectra (d) XPS spectra of Zn.


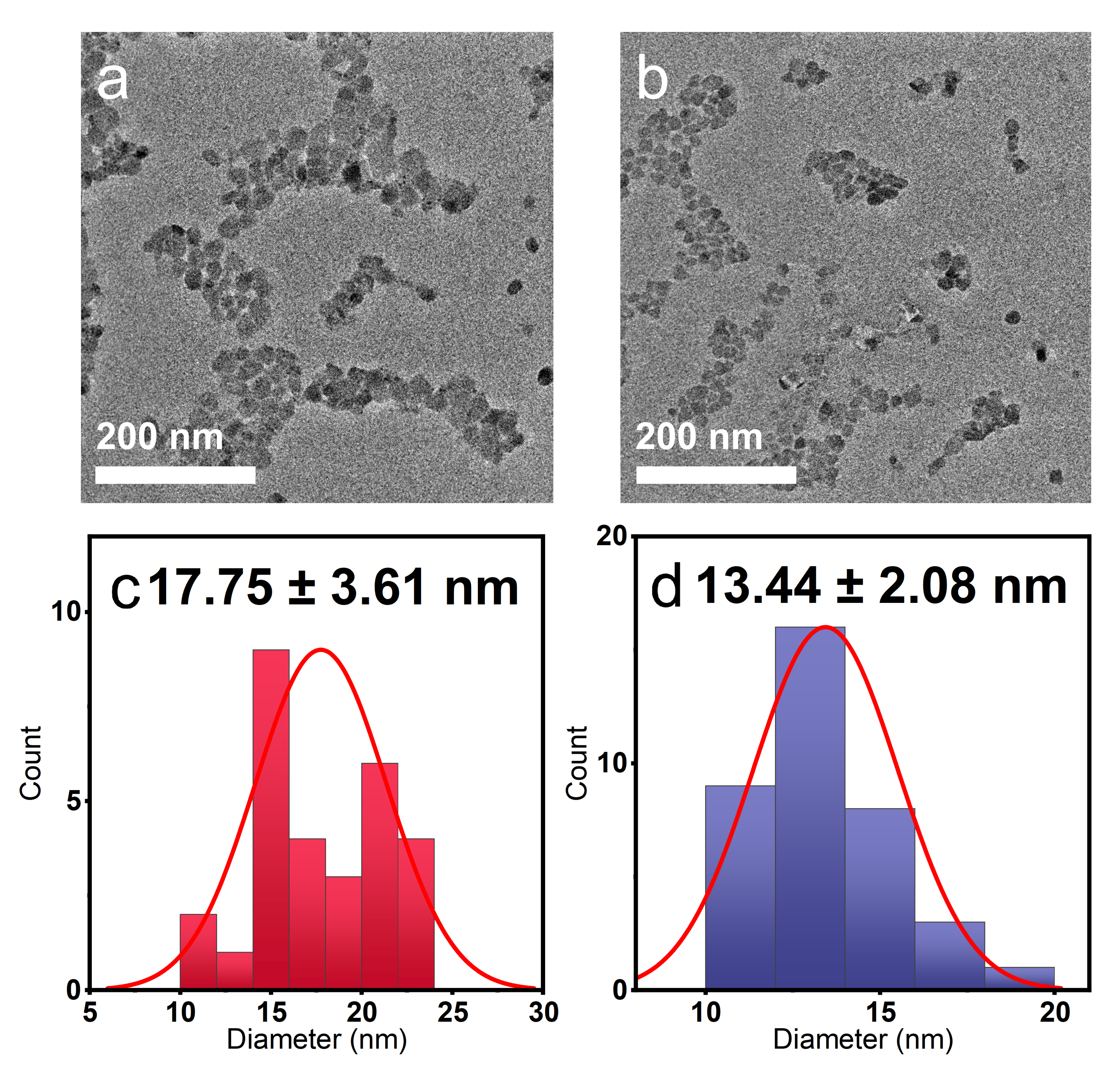


Figure S11. (a-b) TEM images, (c-d) particle size distribution histograms of Cs_3_BiBr_6_ NCs transfer to Cs_4_ZnBi_2_Br_12_ NCs (10 min, 20 min, respectively).


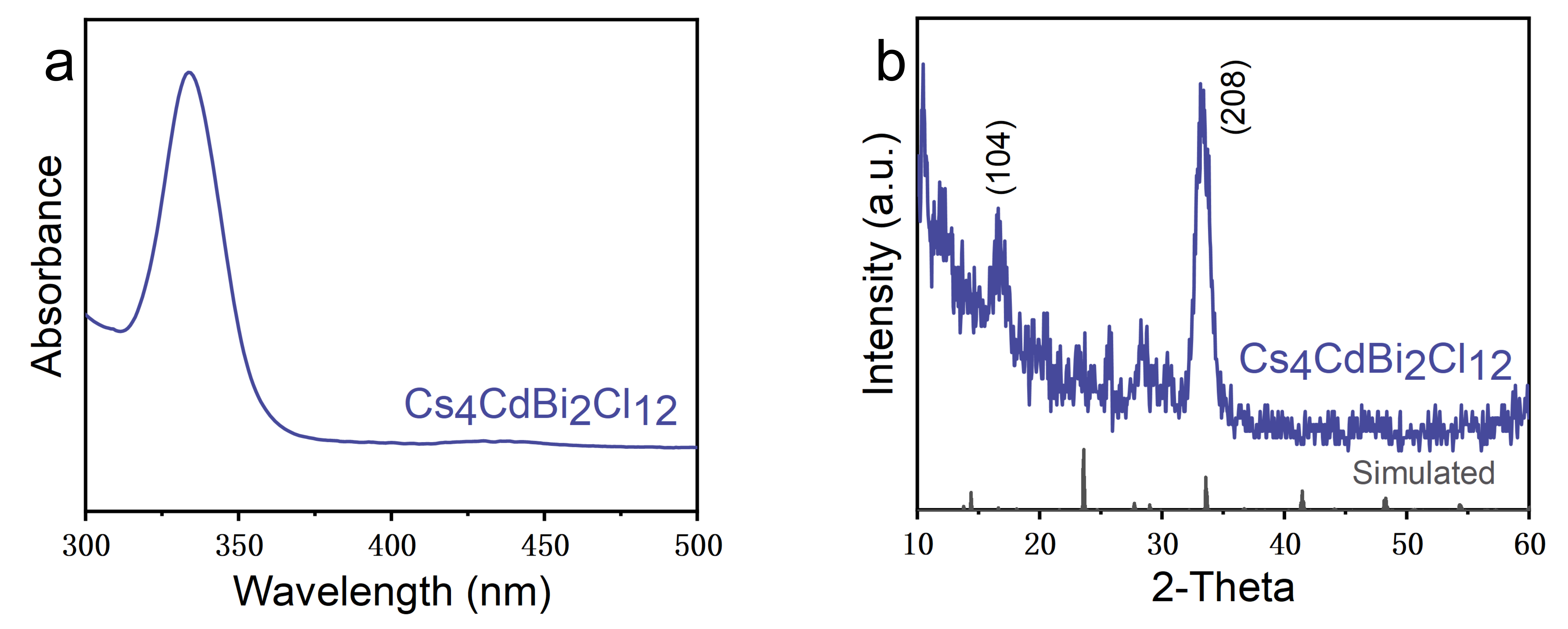


Figure S12. (a) Absorbance, (b) XRD of Cs_4_CdBi_2_Cl_12_ NCs.
